# Correlation among clinical, functional and morphological indexes of the respiratory system in non-cystic fibrosis bronchiectasis patients

**DOI:** 10.1371/journal.pone.0269897

**Published:** 2022-07-06

**Authors:** Jéssica Perossi, Marcel Koenigkam-Santos, Larissa Perossi, Daniele Oliveira dos Santos, Letícia Helena de Souza Simoni, Hugo Celso Dutra de Souza, Ada Clarice Gastaldi

**Affiliations:** 1 Department of Health Sciences, Graduate Program in Functional Performance, Ribeirão Preto Medical School, Ribeirão Preto, São Paulo, Brazil; 2 Department of Medical Imaging, Hematology and Clinical Oncology, Ribeirão Preto Medical School, Ribeirão Preto, São Paulo, Brazil; Clinic for Infectious and tropical diseases, Clinical centre of Serbia, SERBIA

## Abstract

**Background:**

Non-cystic fibrosis bronchiectasis (NCFB) is a heterogeneous disease, which assessment and severity can’t be defined by one particular instrument but using a multidimensional score. Thus, in additional to traditional methods, alternative tools have been developed to assist these patients’ evaluation.

**Objective:**

To correlate functional and morphological indexes with severity and dyspnea in NCFB patients, focusing on the correlation between the impulse oscillometry system (IOS) and the quantitative analysis of computed tomography (CT).

**Methods:**

Clinically stable NCFB patients, between 18 and 80 years old were submitted to clinical, functional and morphological evaluations assessed by Bronchiectasis Severity Index (BSI) and Medical Research Council (MRC) scale; spirometry and IOS; and subjective and quantitative Chest CT scans analysis, respectively.

**Results:**

This study included 38 patients. The best correlations obtained between functional and morphological airway indexes were: resistance at 5 Hz—R5 and the normalized thickness of bronchial walls—Pi10 (r = 0.57), and the mean forced expiratory flow (FEF_25-75%_) and CT score (r = -0.39). BSI as well as MRC showed higher correlations with the quantitative automated analysis of CT (BSI and Pi10: r = 0.41; MRC and Pi10: r = 0.35) than with subjective CT score (BSI and CT score: r = 0.41; MRC and CT score: r = 0.15); and moderate and weak correlations were obtained on both functional airway indexes (BSI and peripheral airways resistance - R5-R20: r = 0.53; BSI and forced expiratory volume at the first second—FEV_1_: R = -0,64; MRC and R5-R20: r = 0.42; and MRC and VEF_1_: r = -0.45).

**Conclusion:**

In NCFB patients, compartmentalized methods for assessing the respiratory system (IOS and the automated quantitative CT analysis) have a good correlation with severity and dyspnea.

## Introduction

Bronchiectasis is a long-term condition with abnormal and permanent dilation of bronchi and bronchioles due to a chronic inflammatory process in these structures [1–4]. Non-Cystic fibrosis bronchiectasis (NCFB) is a heterogeneous condition and the severity of symptons and pulmonary damage can vary widely [5].

Because of this heterogeneity and the different etiologies, it is difficulty to assess the severity of this condition, and different multidimensional severity indexes have been developed to stratify patients’ risk, such as Bronchiectasis Severity Index (BSI), FACED score and E-FACED score [6–8], being the BSI the most widely used.

In addition to conventional modalities for assessing respiratory system morphology and lung function (CT scans and spirometry), alternative methods have been developed in order to provide a more complete and detailed analysis of the respiratory system.

The impulse oscillometry system (IOS) is a tool used to measure pulmonary function that, unlike spirometry, analyzes airways mechanicals properties and lung parenchyma in a non-invasive way and with non-effort dependent maneuvers [9,10]. It is able to identify early changes in the respiratory system and present data related to central and peripheral airways in a compartmentalized way [11–13].

CT scans are used for bronchiectasis diagnosis and follow-up. There are different methods used to quantify the morphology and extension of the bronchiectasis, which are subjective and evaluator’s experience dependent, such as the modified Reiff score. More recently, quantitative analysis programs have been used to study thoracic diseases, seeking more objective and reproductible methods of analyzing medical images [6,14]. Yacta is a fully automatic program, capable of analyzing lung parenchyma, vasculature and airways, obtaining measurements in a three-dimensional and compartmentalized way [15].

Considering the recent advances to assess the respiratory system as well as the heterogeneity of the bronchiectasis, the aim of this study was to correlate functional and morphological indexes with severity and dyspnea in NCFB patients, focusing on the correlation between IOS and the quantitative analysis of CT.

## Methods

This was a cross-sectional study, approved by the Human Research Ethics Committee of the Ribeirao Preto Medical School Clinical Hospital, University of Sao Paulo (CAAE: 36855614.9.0000.5440). All subjects were verbally informed about the study protocol and signed a consent form in a written format.

Subjects were recruited from a convenience sample of patients who were in clinical follow-up at the Pulmonology Outpatient Clinic of Ribeirao Preto Medical School Clinical Hospital, University of Sao Paulo, and were included according to the following inclusion criteria: both genders, between 18 to 80 years old, NCFB diagnosis without exacerbation in the four weeks prior to the evaluations. Patients with cardiovascular diseases were excluded.

Sociodemographic and anthropometric data were collected, the Medical Research Council Dyspnea Scale (MRC) was evaluated. Then volunteers underwent pulmonary function tests–IOS and spirometry—at the Laboratory of Respiratory Assessment in Ribeirao Preto Medical School, University of São Paulo. Every patient was submitted to a chest CT exam in the institution, using the same image acquisition protocol.

### Bronchiectasis Severity Index (BSI)

The BSI incorporates nine variables: age, body mass index (BMI), %FEV_1_ predicted, hospital admission in the preceding two years, exacerbations in the previous year, MRC, *pseudomonas aeruginosa* colonization, colonization with other microorganisms and radiological extension (chest CT) [6]. The total score was calculated by summing the scores for each variable, ranging from 0 to 26 points. According to the overall score, patients are classified into three classes: low BSI score (0–4 points), intermediate BSI score (5–8 points), high BSI score (≥ 9 points).

### Medical Research Council Scale (MRC)

The severity of dyspnea was graduated according to the MRC breathlessness scale into 5 grades: 1 (patient is not troubled by breathlessness except on strenuous exercise); 2 (getting short breath when hurrying on the level or walking up a slight hill); 3 (walking slower than most people on the level, stopping after a mile or so, or stopping after 15 minutes walking at own pace); 4 (stopping for breath after walking about 100 yds or after a few minutes on level ground) and, 5 (being too breathless to leave the house or being breathless when undressing) [16].

### Spirometry

Spirometry was conducted by the Jaeger IOS equipment (Jaeger Wurzburg, Germany), software Launch SentrySuite TM version 2.11, daily calibrated and the maneuvers were performed according to the ATS/ERS statement [17]. The reference equation was the one developed by Crapo et al. (1981) and the analyzed variables were: forced vital capacity (FVC), forced expiratory volume at the first second (FEV_1_), Tiffeneau index (FEV_1_ / FVC) and the mean forced expiratory flow (FEF_25-75%_) [18].

### Impulse Oscillometry System (IOS)

**Fig 1:**
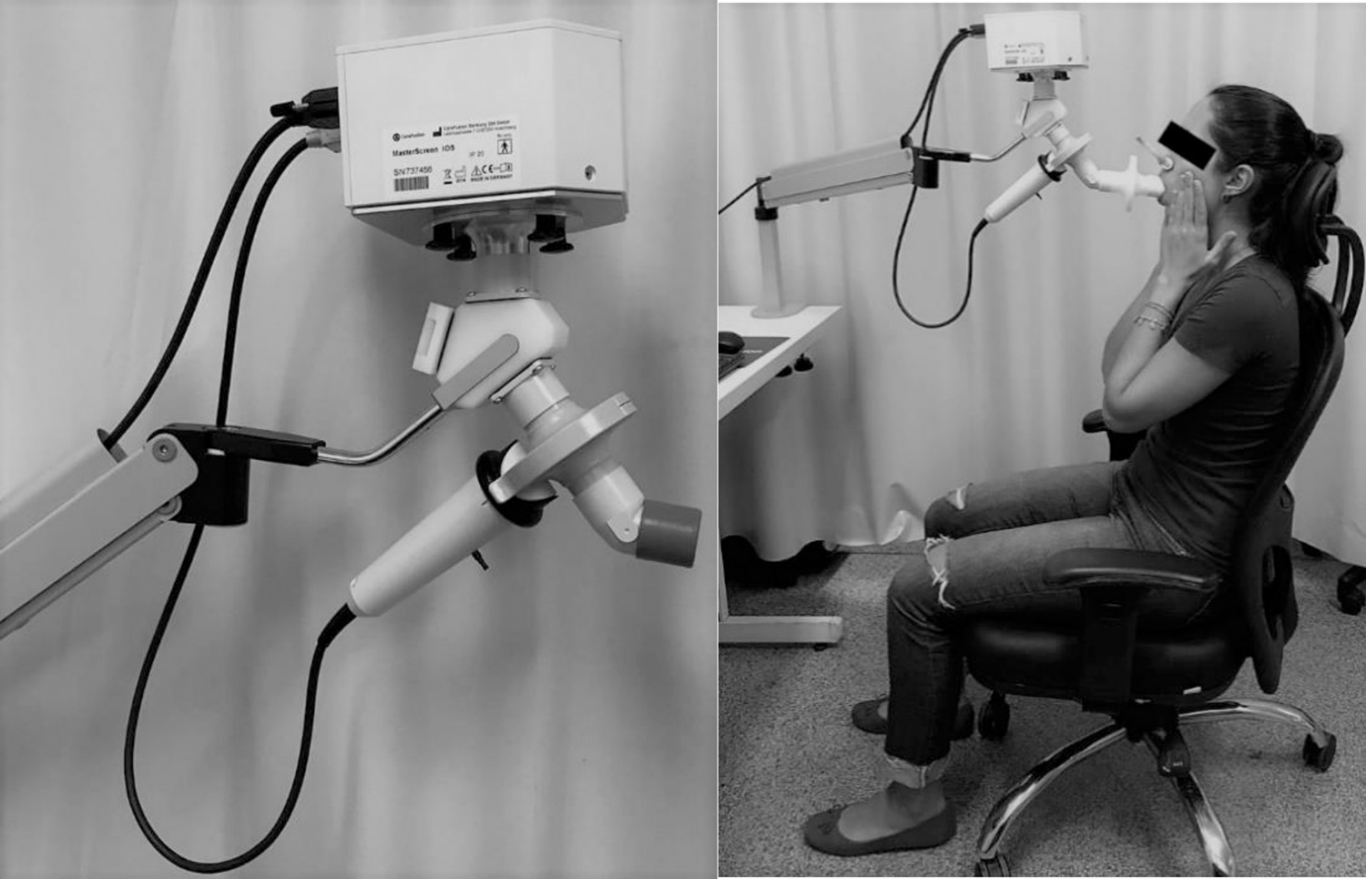
Jaeger IOS equipment (Jaeger, Wurzburg, Germany) and exam positioning.

Impulse oscillometry was assessed by using a Jaeger IOS (Jaeger, Wurzburg, Germany), software Launch SentrySuite TM version 2.11 (Fig 1). The test was performed during spontaneous breathing, with the subjects in a seated position, head in neutral position wearing a nose clip, hands supporting the cheeks to decrease their oscillation and lips tightly sealed around the mouthpiece to avoid air leaks (Fig 1) [19]. A free-flow mouthpiece was used in order to stabilize the position of their tongue [20]. Evaluated parameters included: resistance at 5 Hz and 20 Hz (total airway resistance: R5 and central airway resistance: R20, respectively) and the difference between R5 and R20 (peripheral airway resistance: R5-R20).

### Chest computed tomography (CT)

All CT examinations were performed in a 16-detector MDCT scanner (Brilliance Big Bore; Philips, Amsterdam, the Netherlands). It was applied the high-resolution CT technique, without intravenous administration of iodinated contrast, scanning across the chest in the caudocranial direction, with volumetric acquisition of 1-mm slices at full inspiration. Other typical parameters of acquisition were a tube voltage of 120 kVp; a reference tube current of 110–130 mAs; a gantry rotation time of 0.3–0.7 s; and an ideal exposure to radiation of < 5 mSv. Volumetric acquisitions were reconstructed with a soft/standard filter and with a hard filter, with 1-mm slices and a 1-mm reconstruction interval.

### Subjective CT score

Radiological severity of bronchiectasis was assessed using a modified Reiff score, which assesses the number of lobes involved in the disease (with the lingula considered to be a separate lobe) and the degree of bronchial dilatation (tubular = 1, varicose = 2, and cystic = 3). The maximum score is 18 and minimum score is 1, and it was analyzed by an experienced chest radiologist doctor.

### Quantitative analysis of CT

Quantitative analysis of the respiratory system on CT images were performed using Yacta program, version 2.0, installed on a computer in the image processing lab of our department and connected to the network servers of the hospital [15]. Therefore, the images are sent directly from the scanner to be analyzed by the program.

**Fig 2:**
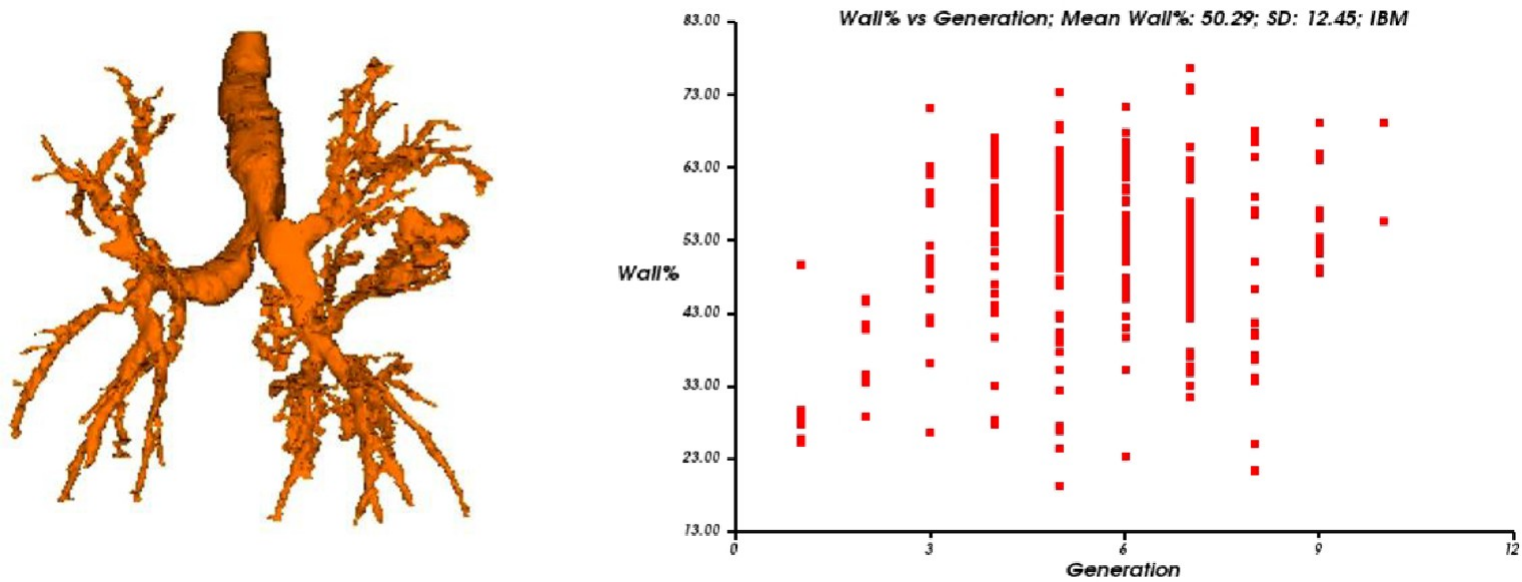
Automated quantitative CT analysis (Yacta program) example of fine included patient. The tracheobronchial tree segmentation with bronchiectasis. (B) a relative bronchial wall thickness measurement (wall%) dispersion graphic is illustrated according to the bronchial generations.

The Yacta program works automatically, not requiring user’s intervention at any stage of the process. Initially, Yacta segments (i.e., anatomically separates) the airways, the right lung, the left lung, and the lung lobes, based on a computerized algorithm for structures recognition, densities, and anatomical thresholds. A center line is calculated, within the airway, from the trachea to the distal bronchi and is used as a reference for measurements in the true transverse plane of the bronchus (perpendicular to the line axis) (Fig 2) [15]. The airway measurements obtained by Yacta are the number of bronchi analyzed; the overall airway; luminal area; wall thickness; relative wall thickness (RWT) of the bronchus; RWT of the third to eighth bronchial generation; normalized thickness of bronchial walls (Pi10); and maximum attenuation of the bronchial wall. We used the following indexes in our analysis: luminal area of the third bronchial generation (AL3), luminal area of the fourth bronchial generation (AL4) and Pi10.

### Statistical analysis

Data were analyzed by using the statistical programs R (R Core Team, Vienna, Austria) and SPSS (version IBM statistic 22.0; SPSS Inc.; Chicago, IL, USA). Correlations between airway measurements (Yacta) and functional parameters (IOS and spirometry) were performed using Pearson’s correlation coefficient. Correlations were interpreted as weak (0 to 0.5), moderate (0.5 to 0.7) or strong (from 0.7 to 1). The level of statistical significance was considered when *p* < .05.

## Results

Thirty-eight NCFB patients were included. The main clinical and functional data of the volunteers are shown in Table 1.

**Table 1:** Clinical and functional data (n = 38).

**Variables**	
**Gender (M/W)**	17/21
**Age (years)**	51.82 ± 15.6
**BMI (kg/m^2^)**	24.41 ± 4.7
**MRC**	2.5 ± 1.06
**BSI Score**	5.58 ± 3.7
**Spirometry**	**L (%)**
FEV_1_	1.74 ± 0.83 (61.1 ± 24.4)
**FVC**	2.75 ± 1.03 (78.2 ± 22.7)
FEV_1_/FVC	0.63 ± 0.14 (76.3 ± 16.3)
FEF_25-75%_	1.14 ± 0.98 (38.5 ± 30.7)
**Impulse Oscillometry System**	**kPa/L/s (%)**
**R5**	0.49 ± 0.19 (147.6 ± 67.7)
**R20**	0.33 ± 0.09 (116.7 ± 33.8)
**R5-R20**	0.16 ± 0.14 (339.7 ± 314.2)

Values expressed by mean±sd. M: Men, W: Women, BMI: body mass index, BSI: Bronchiectasis severity index. FEV_1_: Forced expiratory volume in the first second, FVC: Forced vital capacity, FEV_1_/FVC: Tiffeneau index, FEF_25-75%_: Mean forced expiratory flow. R5: Resistance at 5 Hz, R20: Resistance at 20 Hz.

The CT scans analysis results performed by Yacta and the subjective CT score are shown in Table 2.

**Table 2:** Bronchiectasis subjects CT scans data.

**CT quantitative analysis (Yacta)**	
**AL3**	59.711 ± 28.35
**AL4**	31.054 ± 11.47
**Pi10**	0.3815 ± 0.167
**Wall (%)**	51.647 ± 6.361
**Wall (%) 3,8**	46.439 ± 15.349
**NB**	52.260 ± 35.320
**Subjective CT score**	7.32 ± 3.71

Values expressed by mean±sd. AL3: Luminal area of third bronchial generation, AL4: Luminal area of fourth bronchial generation, Pi10: Normalized thickness of bronchial walls, Wall(%): Relative bronchial wall thickness in percentage, Wall(%)3;8: Relative bronchial wall thickness of third to eighth bronchial generation, NB: Number of bronchi, CT: Computed tomography.

### Quantitative analysis of CT and spirometry

The Yacta program was able to segment and analyze the CT scans of all patients.

AL4 showed statistically significant correlations only with %FEV_1_ (r = 0.36) and CVF (0.34); and Pi10 showed significant weak and moderate correlations with all spirometry parameters, except with %FEF_25-75%_.

### Quantitative analysis of CT and IOS

R5, R5%, R20, R20% and R5-R20 showed statistically significant negative correlations with AL3 (luminal area of the third bronchial generation) and AL4 showed weak and significant negative correlations with R5, %R5 and R20, %R20. (Table 3).

**Table 3:** Correlation between functional and morphological indexes of the respiratory system.

	CT quantitative analysis (Yacta)						Subjective CT Score	
	AL3		AL4		Pi10			
**Espirometria**	**r**	***p* value**	**r**	***p* value**	**r**	***p* value**	**r**	***p* value**
FEV_1_ (L)	0.29	0.076	0.08	0.621	-0.48*	**0.002**	-0.38*	**0.016**
FEV_1_ (%)	0.24	0.137	0.34*	**0.033**	-0.41*	**0.009**	-0.36*	**0.025**
FVC (L)	0.30	0.061	0.04	0.812	-0.42*	**0.009**	-0.32*	**0.045**
FVC (%)	0.21	0.194	0.36*	**0.028**	-0.35*	**0.031**	-0.38*	**0.017**
FEV_1_/FVC (L)	0.13	0.416	0.16	0.334	-0.32*	**0.047**	-0.16	0.319
FEV_1_/FVC (%)	0.19	0.247	0.22	0.182	-0.34*	**0.036**	-0.05	0.740
FEF_25-75%_ (L)	0.19	0.248	0.09	0.575	-0.40*	**0.013**	-0.39*	**0.013**
FEF_25-75%_ (%)	0.21	0.195	0.24	0.132	-0.31*	**0.053**	-0.20	0.223
**IOS**								
R5 (kPa/L/s)	-0.51*	**0.0008**	-0.33*	**0.041**	0.57*	**0.0001**	0.08	0.632
R5 (%)	-0.38*	**0.016**	-0.36*	**0.028**	0.46*	**0.0033**	0.02	0.873
R20 (kPa/L/s)	-0.54*	**0.0004**	-0.36*	**0.026**	0.32	0.0518	-0.25	0.125
R20 (%)	-0.42*	**0.007**	-0.44*	**0.006**	0.28	0.0899	-0.27	0.097
R5-R20 (kPa/L/s)	-0.35*	**0.027**	-0.22	0.179	0.57*	**0.0001**	0.27	0.099
R5-R20 (%)	-0.28	0.087	-0.20	0.222	0.50*	**0.0014**	0.26	0.110

AL3: Luminal area of third bronchial generation, AL4: Luminal area of fourth bronchial generation, Pi10: Normalized thickness of bronchial walls. FEV_1_: Forced expiratory volume in the first second, FVC: Forced vital capacity, FEV_1_/FVC: Tiffeneau index, FEF_25-75%_: Mean forced expiratory flow. R5: Resistance at 5Hz, R20: Resistance at 20 Hz.

*p < .05.

Statistically significant positive correlations were observed between Pi10 and R5, R5%, R20, R5-R20 and %R5-R20. (Table 3).

For the remaining parameters of the quantitative analysis of CT, no significant correlations were found.

### Subjective CT score and spirometry

The subjective CT score for bronchiectasis was negatively correlated with FEV_1_, %FEV_1_, FVC, %FVC and FEF_25-75%_. There were no significant correlations with FEV_1_/FVC, %FEV_1_/FVC and %FEF_25-75%_ (Table 3).

### Subjective CT score and IOS

There were no statistically significant correlations between the subjective CT score for bronchiectasis and IOS parameters (Table 3).

### BSI and functional indexes of the respiratory system

BSI showed statistically significant negative correlations with all evaluated spirometry parameters (Table 4). Statistically significant positive correlations were observed between BSI and peripheral airway resistance indexes (R5-R20; %R5-R20) and with R5.

**Table 4:** Correlation among BSI and MRC and functional and morphological indexes of the respiratory system.

Spirometry	BSI		MRC	
	r	*p* value	r	*p* value
FEV_1_ (L)	-0.64*	**0.00001**	-0.45*	**0.004**
FEV_1_ (%)	-0.54*	**0.0004**	-0.41*	**0.010**
FVC (L)	-0.56*	**0.0002**	-0.40*	**0.012**
FVC (%)	-0.45*	**0.0038**	-0.32*	**0.044**
FEV_1_/FVC	-0.40*	**0.0109**	-0.29	0.072
FEV_1_/FVC (%)	-0.34*	**0.0312**	-0.33*	**0.037**
FEF_25-75%_ (L)	-0.55*	**0.0004**	-0.35*	**0.028**
FEF_25-75%_ (%)	-0.37*	**0.020**	-0.32*	**0.047**
**Impulse Oscillometry System**				
R5 (kPa/L/s)	0.42*	**0.007**	0.38*	**0.018**
R5 (%)	0.27	0.098	0.29	0.077
R20 (kPa/L/s)	0.07	0.651	0.14	0.392
R20 (%)	-0.06	0.681	0.06	0.687
R5-R20 (kPa/L/s)	0.53*	**0.0005**	0.42*	**0.007**
R5-R20 (%)	0.44*	**0.0049**	0.36*	**0.026**
**Morphological indexes**				
AL3	-0.30	0.066	-0.15	0.340
AL4	-0.17	0.303	-0.27	0.098
Pi10	0.41*	**0.0088**	0.35*	**0.029**
Subjective CT score	0.41*	**0.0100**	0.15	0.365

FEV_1_: Forced expiratory volume at the first second, FVC: Forced vital capacity, FEV_1_/FVC: Tiffeneau index, FEF_25-75%_: Mean forced expiratory flow. R5: Resistance at 5Hz, R20: Resistance at 20 Hz. AL3: Luminal area of third bronchial generation, AL4: Luminal area of fourth bronchial generation, Pi10: Normalized thickness of bronchial walls.

*p < .05.

### BSI and morphological indexes of the respiratory system

A statistically significant positive correlation was observed between Pi10 and BSI (Table 4). Between the subjective CT score for bronchiectasis and BSI, a statistically significant positive correlation was demonstrated.

### MRC and functional indexes of the respiratory system

All spirometric data analyzed showed statistically significant negative correlations with MRC, except FEV_1_/FVC (L). With IOS, significant correlations were observed only with R5 and with peripheral airways resistance indexes (R5-R20; %R5-R20).

### MRC and morphological indexes of the respiratory system

Statistically significant correlation was found between MRC and Pi10. There was no statistically significant correlation between MRC and the subjective CT score.

## Discussion

The present study correlated functional and morphological indexes with severity and dyspnea in NCFB patients, focusing on the correlation between IOS and the quantitative analysis of CT, because the use of this exams is not common in the clinics. The severity analysis (BSI) and dyspnea index showed good correlations with functional airway indexes (IOS and spirometry), while the severity index correlated with both CT analysis (subjective and quantitative analysis) and MRC correlated only with the automated quantitative way. It was possible to identify which IOS variables have a better correlation with morphological indexes of the respiratory system than spirometry, and that the more detailed respiratory system evaluation methods, such as the IOS and the automated quantitative analysis of CT, have a better correlation with each other.

Currently, the most suitable imaging method to assist in bronchopulmonary diseases diagnosis and monitoring is CT, which in addition to early detect the development of respiratory system conditions, it allows a compartmentalized and regional assessment of the lungs, being able to evaluate even the presence of bronchiectasis, which present heterogeneous damage that affect central and peripheral airways [21–24]. There are several scores described in the literature for analysis of CT images, and the Reiff score is one of few developed especially for bronchiectasis subjects [14,25,26]. On the other hand, more objective and detailed, automated tools have been developed [15,22].

In this study, we analyzed 38 CT scans of NCFB patients using the most frequently used, the subjective CT score (Reiff score), and an automated quantitative analysis (Yacta program). For pulmonary function, we analyze the commonly used test, the spirometry, considered the gold standard method for functional airways assessment, and the IOS, that can promote a detailed assessment of the respiratory system, providing measurements during the whole breath or during inspiratory or expiratory phase, of central and peripheral airways [11,13].

The subjective CT score was correlated with the analyzed parameters of spirometry, and no statistically significant correlations were obtained with IOS data, which is different from the study of Guan et al. (2016), that found statistically significant correlations between the subjective CT score (modified Reiff score) both with spirometric (% FEF_25-75%_: r = -0.58) and IOS parameters (R5-R20: r = 0.48) [27]. However, it is possible to observe an agreement between both studies, demonstrated that the subjective CT score is a tool that better correlates with spirometry than with more detailed functional analysis of the respiratory system obtained by IOS [27], suggesting that, because they are simplified methods, the subjective CT score and spirometry correlate better with each other.

Regarding the Yacta program, the variables analyzed with the greatest statistical significance correlations were related to segmental bronchial luminal area and to the normalized thickness of the bronchial walls (AL3, AL4 and Pi10). This quantitative analysis of CT tools has been used in previous studies to analyze patients with different bronchopulmonary diseases, however, so far, studies using similar tools to analyze bronchiectasis subjects CT scans were performed only in the disease resulting from cystic fibrosis [22,28,29].

In our study, we correlated the Yacta parameters with spirometric and IOS data, and it was possible to observe statistically significant correlations between the luminal area measurements (3^rd^ and 4^th^ generation bronchial) with almost all IOS indexes, while there was only a weak correlation between AL4 and FEV_1_% and FVC%. The normalized bronchial wall thickness (Pi10) correlated with all spirometric parameters and R5 and R5-R20 in absolute values ​​and as a percentage of predicted. It needs to be noted that the correlations between central and peripheral resistance, from IOS, was stronger than with spirometry. This result can be attributed to the greater detail of the respiratory system assessment that both of these tools (Yacta and IOS) allow.

Some studies that correlated CT quantitative parameters with pulmonary function tests in cystic fibrosis subjects are available in the literature [22,28]. Unlike our results, Koenigkam-Santos et al. (2016) observed that %FEV_1_ was not related to the luminal area or to the relative bronchial wall thickness indicator in subjects without severe airflow obstruction, result that corroborated with the findings of Wielputz et al. (2013) [22,28]. We speculate that these differences within the findings of our study in bronchiectasis patients can be explained by pathophysiological differences of the evaluated diseases.

The correlations of BSI and MRC with functional indexes of the respiratory system of bronchiectasis patients suggests similar information about the disease when they were correlated with spirometry and IOS. The better correlations obtained with spirometric parameters were already expected, because dyspnea and FEV_1_ are domains included in BSI calculation, however, it is important to note that moderate correlations were also demonstrated with IOS parameters. Corroborating to our findings, the study conducted by Guan et al. (2016) observed significant correlations when correlating BSI with %FEF_25-75%_ (r = -0.33) and R5-R20 (r = 0.32), but it is worth mentioning that our study demonstrated a moderate correlation between BSI and R5-R20 (r = 0.53) [27]. This correlation may be related to the fact that our study included subjects classified with severe bronchiectasis by BSI (BSI ≥ 9), while the severe disease was excluded in the Guan and collaborators’ study [27].

The correlations of BSI and MRC with CT analysis were considered weak, but significant from BSI with both Pi10 and subjective CT score, while the dyspnea was correlated only with Pi10, suggesting that the quantitative analysis of CT may be a more efficient tool to relate clinical symptoms of bronchiectasis patients. Besides the correlations were significant of BSI and MRC with IOS and spirometry, the correlation between resistance and flow makes more sense with the mechanism of the disease, specially the central and peripheral resistance and the normalized thickness of the bronchial walls, and to our knowledge, this is the first study that correlated a clinical symptom and severity with functional and morphological indexes, in different levels of NCFB severity.

Once the IOS evaluation and the quantitative analysis of CT are considered the most complex and detailed tools able to assess the respiratory tract, these methods can be more sensitive to detect early changes in this system; therefore, these instruments may have been more effective in indicating these changes in milder levels of severity patients than in subjects who already had major dysfunctions installed.

Thus, we conclude that in NCFB patients, compartmentalized methods for assessing the respiratory system (IOS and the automated quantitative CT analysis) have a good correlation with severity and dyspnea. Therefore, this study reinforces the potential role of these tools for a more detailed and complete airway analysis in the assessment and management of bronchiectasis patients.

## Supporting information

S1 Fig (TIF)

S2 Fig (TIF)

S1 Table Clinical and functional data (n = 38). (PDF)

S2 Table Bronchiectasis subjects CT scans data. (PDF)

S3 Table Correlation between functional and morphological indexes of the respiratory system. (PDF)

S4 Table Correlation among BSI and MRC and functional and morphological indexes of the respiratory system. (PDF)
