# Supplementary figures and images for: Correlation among clinical, functional and morphological indexes of the respiratory system in non-cystic fibrosis bronchiectasis patients
Source: PLoS One. 2022 Jul 6;17(7):e0269897. doi: 10.1371/journal.pone.0269897 (PMC9258820; doi:10.1371/journal.pone.0269897)

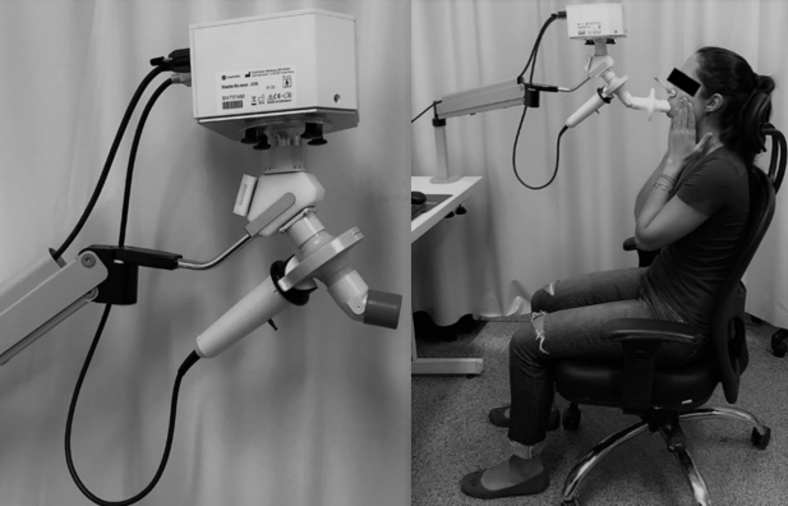

Supplement: S1 Fig — (TIF) [file pone.0269897.s001.tif]

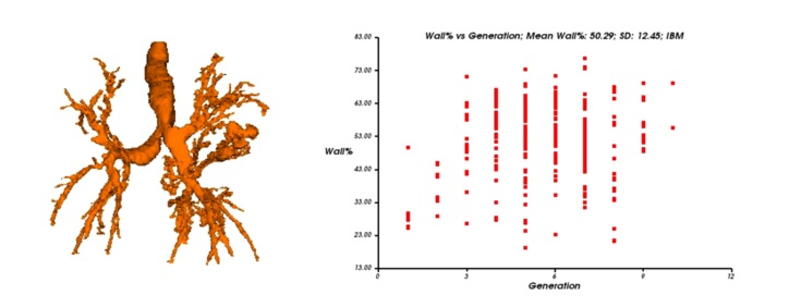

Supplement: S2 Fig — (TIF) [file pone.0269897.s002.tif]
