# Supplementary material for: Correlation among clinical, functional and morphological indexes of the respiratory system in non-cystic fibrosis bronchiectasis patients
Source: PLoS One. 2022 Jul 6;17(7):e0269897. doi: 10.1371/journal.pone.0269897 (PMC9258820; doi:10.1371/journal.pone.0269897)
Supplement: S1 Table — (PDF) [file pone.0269897.s003.pdf]

Table 1. Clinical and functional data (n=38)	
**Variables**	
**Gender (M/W)**	17/21
**Age (years)**	51.82 ± 15.6
**BMI (kg/m²)**	24.41 ± 4.7
**MRC**	2.5 ± 1.06
**BSI Score**	5.58 ± 3.7
**Spirometry**	**L (%)**
**FEV_1**	1.74 ± 0.83 (61.1 ± 24.4)
**FVC**	2.75 ± 1.03 (78.2 ± 22.7)
**FEV_1/FVC**	0.63 ± 0.14 (76.3 ± 16.3)
**$FEF_{25-75\%}$**	1.14 ± 0.98 (38.5 ± 30.7)
**Impulse Oscillometry System**	**kPa/L/s (%)**
**R5**	0.49 ± 0.19 (147.6 ± 67.7)
**R20**	0.33 ± 0.09 (116.7 ± 33.8)
**R5-R20**	0.16 ± 0.14 (339.7 ± 314.2)

Values expressed by mean±sd. M: men, W: women, BMI: body mass index, BSI: bronchiectasis severity index. FEV_1: forced expiratory volume in the first second, FVC: forced vital capacity, FEV_1/FVC: Tiffeneau index, $FEF_{25-75\%}$: mean forced expiratory flow. R5: resistance at 5 Hz, R20: resistance at 20 Hz.
