# Supplementary material for: Correlation among clinical, functional and morphological indexes of the respiratory system in non-cystic fibrosis bronchiectasis patients
Source: PLoS One. 2022 Jul 6;17(7):e0269897. doi: 10.1371/journal.pone.0269897 (PMC9258820; doi:10.1371/journal.pone.0269897)
Supplement: S2 Table — (PDF) [file pone.0269897.s004.pdf]

Table 2. Bronchiectasis subjects CT scans data

**CT quantitative analysis (Yacta)**	
**AL3**	59.711 ± 28.35
**AL4**	31.054 ± 11.47
**Pi10**	0.3815 ± 0.167
**Wall (%)**	51.647 ± 6.361
**Wall (%) 3,8**	46.439 ± 15.349
**NB**	52.260 ± 35.320
**Subjective CT score**	7.32 ± 3.71

Values expressed by mean±sd. AL3: luminal area of third bronchial generation, AL4: luminal area of fourth bronchial generation, Pi10: normalized thickness of bronchial walls, Wall(%): relative bronchial wall thickness in percentage, Wall(%)3;8: relative bronchial wall thickness of third to eighth bronchial generation, NB: number of bronchi, CT: computed tomography.
