# Supplementary material for: Correlation among clinical, functional and morphological indexes of the respiratory system in non-cystic fibrosis bronchiectasis patients
Source: PLoS One. 2022 Jul 6;17(7):e0269897. doi: 10.1371/journal.pone.0269897 (PMC9258820; doi:10.1371/journal.pone.0269897)
Supplement: S3 Table — (PDF) [file pone.0269897.s005.pdf]

Table 3. Correlation between functional and morphological indexes of the respiratory system

	CT quantitative analysis (Yacta)						Subjective CT Score	
	AL3		AL4		Pi10			
**Espirometria**	**r**	***p* value**	**r**	***p* value**	**r**	***p* value**	**r**	***p* value**
**FEV_1 (L)**	0.29	0.076	0.08	0.621	-0.48*	**0.002**	-0.38*	**0.016**
**FEV_1 (%)**	0.24	0.137	0.34*	**0.033**	-0.41*	**0.009**	-0.36*	**0.025**
**FVC (L)**	0.30	0.061	0.04	0.812	-0.42*	**0.009**	-0.32*	**0.045**
**FVC (%)**	0.21	0.194	0.36*	**0.028**	-0.35*	**0.031**	-0.38*	**0.017**
**FEV_1/FVC (L)**	0.13	0.416	0.16	0.334	-0.32*	**0.047**	-0.16	0.319
**FEV_1/FVC (%)**	0.19	0.247	0.22	0.182	-0.34*	**0.036**	-0.05	0.740
**$FEF_{25-75\%}$ (L)**	0.19	0.248	0.09	0.575	-0.40*	**0.013**	-0.39*	**0.013**
**$FEF_{25-75\%}$ (%)**	0.21	0.195	0.24	0.132	-0.31*	**0.053**	-0.20	0.223
**IOS**								
**R5 (kPa/L/s)**	-0.51*	**0.0008**	-0.33*	**0.041**	0.57*	**0.0001**	0.08	0.632
**R5 (%)**	-0.38*	**0.016**	-0.36*	**0.028**	0.46*	**0.0033**	0.02	0.873
**R20 (kPa/L/s)**	-0.54*	**0.0004**	-0.36*	**0.026**	0.32	0.0518	-0.25	0.125
**R20 (%)**	-0.42*	**0.007**	-0.44*	**0.006**	0.28	0.0899	-0.27	0.097
**R5-R20 (kPa/L/s)**	-0.35*	**0.027**	-0.22	0.179	0.57*	**0.0001**	0.27	0.099
**R5-R20 (%)**	-0.28	0.087	-0.20	0.222	0.50*	**0.0014**	0.26	0.110

AL3: luminal area of third bronchial generation, AL4: luminal area of fourth bronchial generation, Pi10: normalized thickness of bronchial walls. FEV_1: forced expiratory volume in the first second, FVC: forced vital capacity, FEV_1/FVC: Tiffeneau index, $FEF_{25-75\%}$: mean forced expiratory flow. R5: resistance at 5Hz, R20: resistance at 20 Hz. *p<.05.
