# Supplementary material for: Correlation among clinical, functional and morphological indexes of the respiratory system in non-cystic fibrosis bronchiectasis patients
Source: PLoS One. 2022 Jul 6;17(7):e0269897. doi: 10.1371/journal.pone.0269897 (PMC9258820; doi:10.1371/journal.pone.0269897)
Supplement: S4 Table — (PDF) [file pone.0269897.s006.pdf]

Table 4. Correlation among BSI and MRC and functional and morphological indexes of the respiratory system

**Spirometry**	**BSI**		**MRC**	
	**r**	***p* value**	**r**	***p* value**
**FEV_1 (L)**	-0.64*	**0.00001**	-0.45*	**0.004**
**FEV_1 (%)**	-0.54*	**0.0004**	-0.41*	**0.010**
**FVC (L)**	-0.56*	**0.0002**	-0.40*	**0.012**
**FVC (%)**	-0.45*	**0.0038**	-0.32*	**0.044**
**FEV_1/FVC**	-0.40*	**0.0109**	-0.29	0.072
**FEV_1/FVC (%)**	-0.34*	**0.0312**	-0.33*	**0.037**
**$FEF_{25-75\%}$ (L)**	-0.55*	**0.0004**	-0.35*	**0.028**
**$FEF_{25-75\%}$ (%)**	-0.37*	**0.020**	-0.32*	**0.047**
**Impulse Oscillometry System**				
**R5 (kPa/L/s)**	0.42*	**0.007**	0.38*	**0.018**
**R5 (%)**	0.27	0.098	0.29	0.077
**R20 (kPa/L/s)**	0.07	0.651	0.14	0.392
**R20 (%)**	-0.06	0.681	0.06	0.687
**R5-R20 (kPa/L/s)**	0.53*	**0.0005**	0.42*	**0.007**
**R5-R20 (%)**	0.44*	**0.0049**	0.36*	**0.026**
**Morphological indexes**				
**AL3**	-0.30	0.066	-0.15	0.340
**AL4**	-0.17	0.303	-0.27	0.098
**Pi10**	0.41*	**0.0088**	0.35*	**0.029**
**Subjective CT score**	0.41*	**0.0100**	0.15	0.365

FEV_1: forced expiratory volume at the first second, FVC: forced vital capacity, FEV_1/FVC: Tiffeneau index, $FEF_{25-75\%}$: mean forced expiratory flow. R5: resistance at 5Hz, R20: resistance at 20 Hz. AL3: luminal area of third bronchial generation, AL4: luminal area of fourth bronchial generation, Pi10: normalized thickness of bronchial walls. *p<.05.
